# Intraspecific Genetic Admixture and the Morphological Diversification of an Estuarine Fish Population Complex

**DOI:** 10.1371/journal.pone.0123172

**Published:** 2015-04-09

**Authors:** Julian J. Dodson, Audrey Bourret, Marie France Barrette, Julie Turgeon, Gaétan Daigle, Michel Legault, Frédéric Lecomte

**Affiliations:** 1 Département de Biologie, Université Laval, Québec, Québec, Canada; 2 Département de Biologie, Université de Sherbrooke, Sherbrooke, Québec, Canada; 3 Département de Mathématique et de Statistique, Université Laval, Québec, Québec, Canada; 4 Direction Générale de l’Expertise sur la Faune et ses Habitats-Secteur de la Faune, Ministère des Forêts, de la Faune et des Parcs, Québec, Québec, Canada; Leibniz-Institute of Freshwater Ecology and Inland Fisheries, GERMANY

## Abstract

The North-east American Rainbow smelt (*Osmerus mordax*) is composed of two glacial races first identified through the spatial distribution of two distinct mtDNA lineages. Contemporary breeding populations of smelt in the St. Lawrence estuary comprise contrasting mixtures of both lineages, suggesting that the two races came into secondary contact in this estuary. The overall objective of this study was to assess the role of intraspecific genetic admixture in the morphological diversification of the estuarine rainbow smelt population complex. The morphology of mixed-ancestry populations varied as a function of the relative contribution of the two races to estuarine populations, supporting the hypothesis of genetic admixture. Populations comprising both ancestral mtDNA races did not exhibit intermediate morphologies relative to pure populations but rather exhibited many traits that exceeded the parental trait values, consistent with the hypothesis of transgressive segregation. Evidence for genetic admixture at the level of the nuclear gene pool, however, provided only partial support for this hypothesis. Variation at nuclear AFLP markers revealed clear evidence of the two corresponding mtDNA glacial races. The admixture of the two races at the nuclear level is only pronounced in mixed-ancestry populations dominated by one of the mtDNA lineages, the same populations showing the greatest degree of morphological diversification and population structure. In contrast, mixed-ancestry populations dominated by the alternate mtDNA lineage showed little evidence of introgression of the nuclear genome, little morphological diversification and little contemporary population genetic structure. These results only partially support the hypothesis of transgressive segregation and may be the result of the differential effects of natural selection acting on admixed genomes from different sources.

## Introduction

Numerous intraspecific phylogeographic studies show how many vertebrate species are composed of morphologically and genetically distinct lineages resulting from the vicariant isolation of intraspecific groups in isolated refugia, particularly those created during Pleistocene continental glaciation events [1]. Historical divergence and subsequent secondary contact of distinct races may result in the genetic admixture of previously isolated gene pools. In such cases, several processes have been hypothesized to occur. On the one hand, if such intraspecific admixture produces hybrids of low fitness, then reinforcement can lead to character displacement and the evolution of reproductive isolation between distinct morphotypes, a process that is the intraspecific analog of interspecific character displacement [2, 3, 4, 5]. On the other hand, such hybridization may produce hybrids that are not less fit, involving either the production of new populations of mixed ancestry that remain distinct from both parental populations (the early stages of hybrid speciation: [6, 7] or the production of a hybrid swarm where the boundaries between the parental and hybrid populations are eventually blurred. According to the model of additive genetic variance, gene flow between divergent allopatric populations will tend to reduce differences between them [8].

Hybridization may also have positive effects on fitness, including heterosis (hybrid vigor) at the individual level and/or high genetic variance for relevant phenotypic traits at the population level [9, 10]. These two features may help hybrids to adapt to novel and/or heterogeneous environments better than the parental lineages [10]. For example, several studies support the hypothesis that invasive organisms from disparate native-range sources form genetically admixed populations with elevated genetic variation that may facilitate invasion success and the displacement of native populations [11,12,13]. Thus, intraspecific hybridization may represent an important mechanism contributing to phenotypic diversification and population formation following the post-glacial recolonization events that bring into contact previously isolated intraspecific races.

Studies of morphological traits in some hybrid populations have reported the presence of extreme phenotypes with trait values exceeding the range of parental trait values, a phenomenon known as transgressive segregation [14, 15]. This is hypothesized to be an important mechanism responsible for producing novel adaptations observed in new hybrid forms [14]. Furthermore, morphological differences among recently founded populations can be explained by the differential admixture of source populations [12]. Thus, contemporary phenotypic variation among intraspecific populations may be attributable to intraspecific hybridization in the current environment.

The rainbow smelt (*Osmerus mordax*), a widespread, abundant, carnivorous fish of northeastern North America occupying lakes, rivers, estuaries and coastal waters, provides an opportunity to assess the consequences of genetic admixture on morphological divergence and population structure. Anadromous/estuarine ecotypes naturally occur from the upper St. Lawrence River to Newfoundland and southwards along the Atlantic seaboard [16]. They form numerous local populations supposedly reflecting historical landscape isolation maintained by low dispersal and hypothesized selective forces [17,18,19]. The species is composed of two glacial races characterized by distinct mtDNA lineages first identified using RFLP over the entire mitochondrial genome, corresponding to a 0.7% net sequence divergence [20,21,22]. Direct sequencing of several mitochondrial genes allowed identifying diagnostic restriction sites [23]. The distribution of the two mtDNA lineages throughout the native range reveal a geographical dichotomy far more evident in lacustrine than estuarine populations. In lakes, populations of the St. Lawrence River drainage are either fixed or largely dominated by one lineage and populations to the south of the Appalachian Mountains are largely dominated by the alternate lineage. Such a dichotomy is far less evident in estuarine populations [22]. This phylogeographic discontinuity strongly suggested that smelt survived in two glacial refugia and followed different post-glacial dispersal routes. The most likely refugium for the more easterly distributed lineage was hypothesized to be the Acadian refugium located on the exundated Grand Banks whereas that of the more westerly-distributed lineage was the Atlantic refugium located along the Atlantic coastal plains [22]. The two mtDNA lineages are thus considered diagnostic for the Acadian and Atlantic races.

Following the retreat of the glaciers, it is hypothesized that smelt from the Atlantic refugium colonized continental areas through the Hudson River valley and colonized the St. Lawrence River watersheds [21,22]. Smelt from the Acadian refuge preferentially colonized the watersheds of the Gulf of St. Lawrence and the Atlantic coast. The great majority of contemporary lacustrine populations along putative post-glacial dispersion routes comprise a single lineage characteristic of either glacial race, although there are rare occurrences of the alternate lineage [22]. Thus, the two mtDNA races may not be completely reciprocally monophyletic because of some retention of ancestral polymorphism. Contemporary breeding populations of estuarine smelt in the St. Lawrence drainage appear unique in comprising contrasting mixtures of both lineages, suggesting that the two glacial races came into secondary contact in the St. Lawrence River [22]. This scenario is consistent with the identification of Lake Ontario and the St. Lawrence River as an important suture zone, encompassing a cluster of secondary contact zones for many northeastern fish species that survived in more than one glacial refugium [24].

Co-occurrence of the two smelt races in contemporary breeding populations along the St. Lawrence River drainage system suggests that smelt derived from the two refugia initially interbred upon secondary contact before forming a number of morphologically and genetically distinct populations [21, 22, 25, 26]. Of particular interest is the observation that two populations occur sympatrically in the brackish water estuary, with one distributed mostly in the channels along the north shore and the second restricted to the shallow shoals of the south shore of the estuary. These two populations are characterized by the presence of both mtDNA lineages but in significantly different frequencies. The so-called north shore population is characterized by the predominance of the Atlantic race (85% of individuals exhibit a mtDNA Atlantic lineage) and the so-called south shore population is characterized by the predominance of the Acadian race (82% of individuals exhibit a mtDNA Acadian lineage). This suggests a period of mixing following secondary contact of the two founding races in the St. Lawrence estuary that subsequently produced two sympatric populations exhibiting distinct patterns of life history, morphology and ecology [27, 28]. Genetic evidence based on nuclear DNA is, however, lacking.

The overall objective of this study was to assess the role of intraspecific genetic admixture in the morphological diversification of estuarine rainbow smelt. Although the two races possess distinct morphologies in the lacustrine environment, these morphologies were observed in populations composed uniquely of one of the two glacial races [29]. The extent of morphological divergence among estuarine populations as a function of differing proportions of the two ancestral races is unknown. Given the strong suggestion of secondary contact between the two races in the St. Lawrence River and the contrasting proportions of the founding races in estuarine populations, we hypothesized that intraspecific genetic admixture may have contributed to phenotypic variation among populations. We first tested if and how the morphology of mixed-ancestry populations (co-occurrence of Acadian and Atlantic mtDNA races) varied as a function of the relative contribution of the two races to estuarine populations sampled throughout northeastern North America. In the case of a significant effect of mixed ancestry on morphological diversity, we tested two alternative outcomes. If gene flow between divergent allopatric populations reduces differences between them, we expected that populations of mixed ancestry would exhibit intermediate morphologies in comparison with populations derived uniquely from either glacial race. Conversely, in the case of transgressive segregation, populations of mixed ancestry would exhibit extreme morphologies relative to populations derived uniquely from either glacial race. Secondly, given the possibility that differential mtDNA lineage composition may be due to the retention of ancestral polymorphism rather than genetic admixture following secondary contact, we sought evidence that two ancestral races are evident within the nuclear gene pool and that the co-occurrence of mtDNA lineages reflects genetic admixture and introgression at the level of the nuclear genome. Finally, nuclear genotype distribution throughout the putative zone of secondary contact is used to test the presumption that the two mtDNA lineages truly represent two divergent glacial races.

## Material and Methods

### General methodology

**Fig 1:**
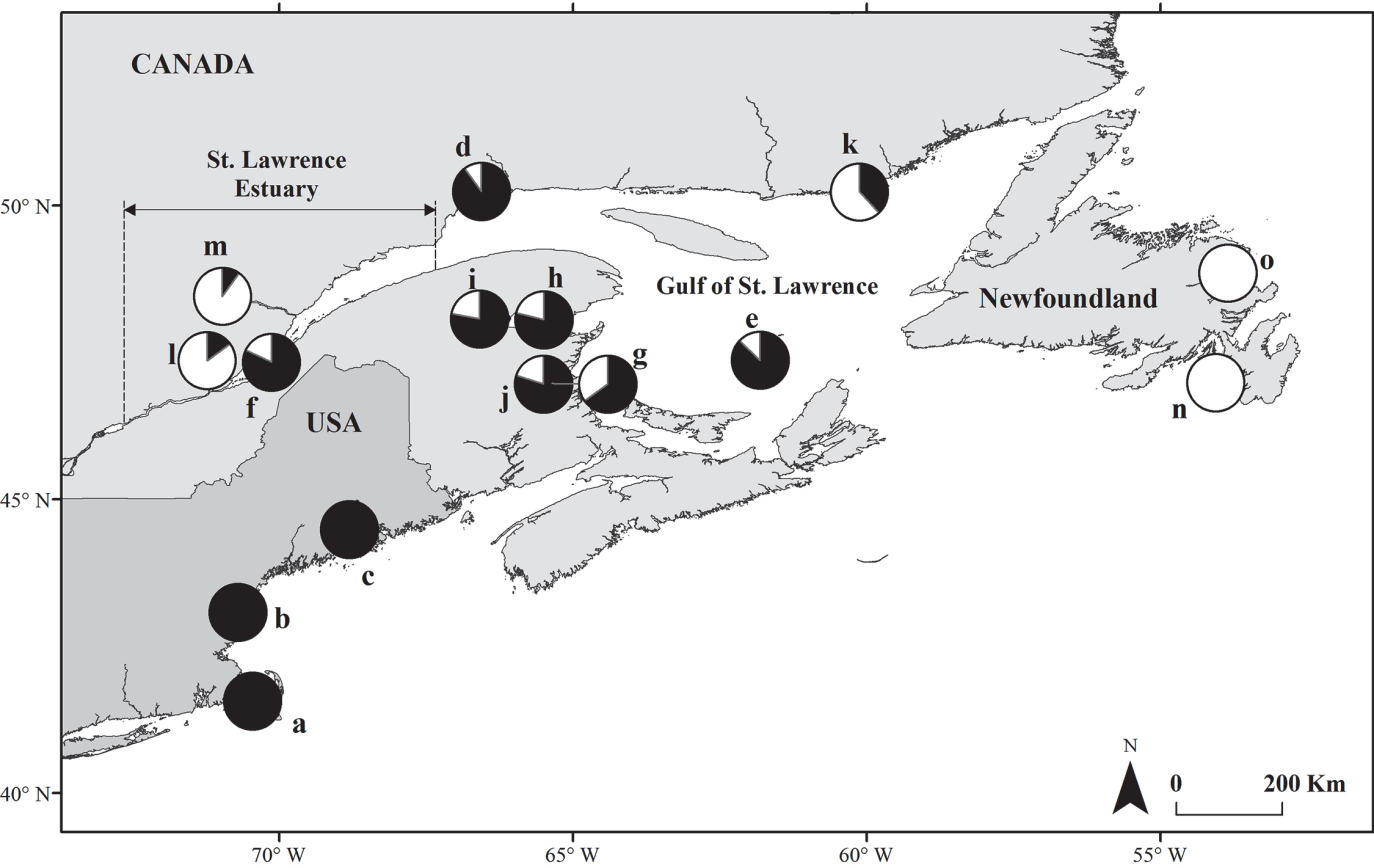
Map illustrating the location of sites in North-east North America sampled to describe the morphological diversity and mtDNA lineage composition of rainbow smelt populations. Pies represent the relative proportions of the two glacial races in each sample (see table 1 for place names, sample sizes and exact proportions). The black sectors represent the proportional representation of the mtDNA Acadian race, the open sectors, the mtDNA Atlantic race.

**Fig 2:**
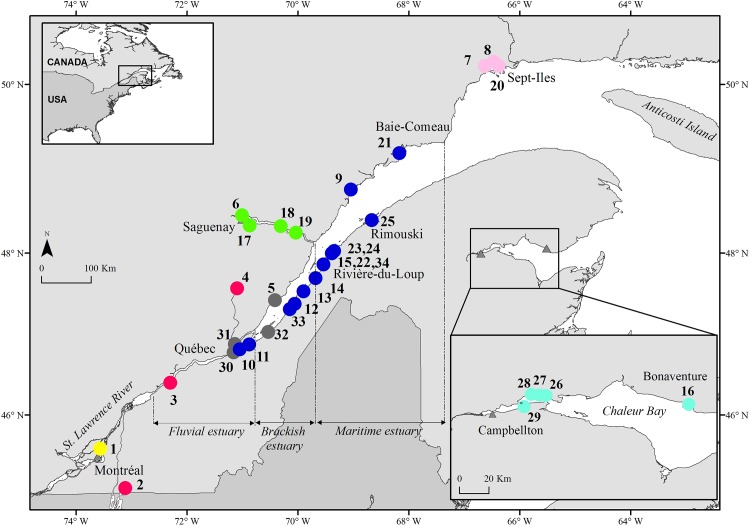
Map illustrating the locations of sites in the St. Lawrence R. and estuary sampled to describe the nuclear DNA genetic structure of rainbow smelt populations encompassing the purported secondary contact zone of the two glacial races. Filled points identify sample sites, color-coded to illustrate the seven genetic populations revealed by the analysis with Structure (see Fig 5). The principal ecological divisions of the St. Lawrence River and estuary are indicated with broken, vertical lines. Pale triangles identify principal towns and cities mentioned in the text. See Table 2 for place names, developmental stage and sample sizes.

In order to achieve our first objective, we exploited a previously published data base consisting of 15 estuarine populations sampled throughout northeastern North America and characterized by a wide variation in the proportional representation of each founding race (Fig 1, Table 1 [30]). In order to achieve our second objective, we conducted a population nuclear genetic analysis of smelt sampled throughout the purported St. Lawrence secondary contact zone (Fig 2).

**Table 1:** Region, sample IDs, sample site locations, and sample sizes for morphological and mtDNA analyses of rainbow smelt. SLR- St. Lawrence River.

Region	Sample ID	Location	Developmental stage	N morpho	N mtDNA	% Acadian
Massachusetts	a	Neponset R.	Adults	43	43	100
New Hampshire	b	Great Bay	Adults	41	41	100
Maine	c	Penobscot R.	Adults	40	40	100
Lower North Shore, SLR	d	Hall R.	Adults	53	53	90
Gulf St. Lawrence	e	Îles de la Madeleine	Adults	40	40	87
South Shore, SLR	f	Ouelle R.	Adults	39	39	82
New Brunswick	g	NW Miramichi R.	Adults	20	20	80
Chaleurs Bay	h	Bonaventure R.	adults	38	38	79
Chaleurs Bay	i	Christopher Brook	Adults	27	27	78
New Brunswick	j	SW Miramichi R.	Adults	20	20	65
Gulf of St. Lawrence	k	St-Augustin	Adults	40	40	38
North Shore, SLR	l	Île aux Coudres	Adults	40	40	15
Saguenay Fjord	m	Chicoutimi	Adults	40	40	10
Newfoundland	n	St. Mary’s Bay	Adults	42	42	0
Newfoundland	o	Gambo R.	Adults	53	53	0

All fish were adults and were sampled between February and September, 2002. Percent composition of mtDNA Acadian lineage from Barrette [29].

The sampling of all life-history stages of fishes during the field part of this study was under the responsibility of Frederic Lecomte and Michel Legault, Ministère des Forêts, de la Faune et des Parcs (MFFP). All fish collection sites were located on public lands and their locations are specified in Table 2. The MFFP is exempt from the requirement of project-specific permits when fish are sacrificed as the result of fishing activity. A waiver of approval was granted by the Comité de protection des animaux, Direction générale de l'expertise sur la faune et ses habitats—Secteur de la faune, Ministère des Forêts, de la Faune et des Parcs (MFFP), Québec (Marcel Bernard, coordonnateur). All fish were frozen upon capture by technicians under the responsibility of FL and ML.

**Table 2:** Sample categories, numerical IDs, sample site locations, dates, developmental stages and sample sizes for AFLP and mtDNA analyses of rainbow smelt.

**Type**	**Region**	**Sample ID**	**Location**	**Developmental stage**	**N AFLP**	**N mtDNA**
**Spawning sites**	Upstream, SLR	1	Montréal	YOY	14	11
		2	Lake Champlain	genitors	12	5
		3	Gentilly	Adults	14	10
		4	Lake Jacques Cartier	Adults	14	5
	North Shore, SLR	5	Île aux Coudres	Adults	10	10
	Saguenay Fjord	6	Chicoutimi	genitors	15	10
	Lower North Shore, SLR	7	Hall R.	genitors	15	12
		8	Du Poste R.	genitors	16	13
		9	Laval R.	genitors	16	6
	South Shore, SLR	10	L’église Brook	genitors	15	3
		11	Boyer R.	genitors	5	4
		12	Ouelle R.	genitors	15	6
		13	Kamouraska R.	YOY	10	3
		14	Fouquette R.	genitors	13	7
		15	Du Loup R.	genitors	13	4
	Chaleurs Bay	16	Bonaventure R.	genitors	10	9
Totals					207	118
**Winter fishery**	Saguenay Fjord	17	Ha Ha Bay	Adults	14	10
		18	Éternité Bay	Adults	14	
		19	St-Jean Cove	Adults	14	
	Lower North Shore, SLR	20	Sept-Îles	Adults	13	
		21	Baie Comeau	Adults	13	
	South Shore, SLR	22	Du Loup R.	Adults	15	
		23	Île Verte channel	Adults	15	
		24	Île Verte R.	Adults	15	
		25	Rimouski Bay	Adults	15	
	Chaleur Bay	26	Miguasha	Adults	14	2
		27	Escouminac Bay	Adults	15	8
		28	Escouminac R.	Adults	6	2
		29	Dalhousie Jct.	Adults	6	1
Totals					169	23
**YOY**	North shore, SLR	30	Québec-Neuville	YOY	14	9
		31	Île d’Orléans	YOY	14	3
	South shore, SLR	32	Montmagny	YOY	15	
		33	Ste-Anne Cove	YOY	14	
		34	Du Loup R.	YOY	14	
Totals					71	12

All fish were sampled during 2011–2012. SLR- St. Lawrence River, YOY- young of the year (larval and post metamorphic stages).

### Populations and morphological diversity

#### Sampling and race assignment

To assign each fish of the 15 anadromous populations to its historical race and thus establish the extent of mixing of the two races at each sampling site, we identified diagnostic mtDNA lineages according to [23]. Three estuarine populations were subsequently assigned uniquely to the Acadian race (n = 124) and 2 were assigned uniquely to the Atlantic race (n = 95). Ten estuarine populations were comprised of both races. Although the two mtDNA lineages may not be fully diagnostic of the two ancestral races, the species’ phylogeographic structure, presented above, strongly suggests that the co-occurrence of the mtDNA lineages in estuarine populations is indicative of admixture of the founding races. As such, we defined 7 admixed populations that were predominantly Acadian in origin (Acadian-mixed, n = 237) and 3 admixed populations that were predominantly Atlantic in origin (Atlantic-mixed, n = 120) (Table 1).

#### Morphological analyses

**Fig 3:**
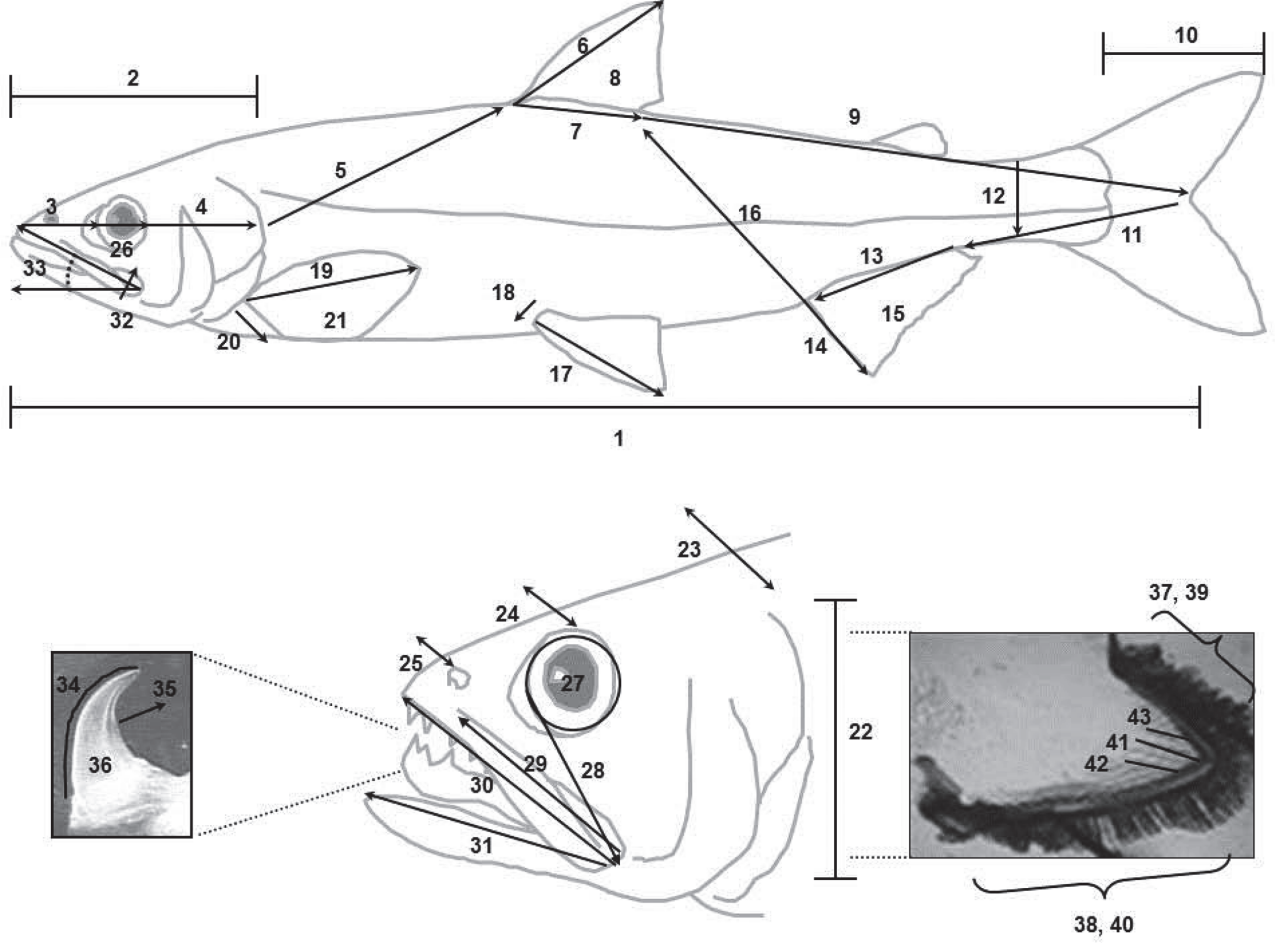
Morphometric and meristic traits measured on 472 Rainbow smelt. 1. Fork length; 2. Head length; 3. Snout length; 4. Post-orbital length; 5. Trunk length; 6. Dorsal fin length; 7. Dorsal fin base; 8. Dorsal fin ray count; 9. Dorsal fin to fork length; 10. Caudal fin length; 11. Fork to anal fin length; 12. Caudal peduncle depth; 13. Anal fin base; 14. Anal fin length; 15. Anal fin ray count; 16. Anal fin to dorsal fin length; 17. Pelvic fin length; 18. Pelvic fin base; 19. Pectoral fin length; 20. Pectoral fin base; 21. Pectoral fin ray count; 22. Head depth; 23. Head width; 24. Inter orbital width; 25. Inter nasal width; 26. Eye diameter; 27. Eye area; 28. Eye to maxillary length; 29. Maxillary length; 30. Upper jaw length; 31. Lower jaw length; 32. Maxillary depth; 33. Maxillary angle; 34. Lingual tooth length; 35. Lingual tooth curve; 36. Large lingual tooth count 37. Dorsal gill arch length; 38. Ventral gill arch length; 39. Dorsal gill raker count; 40. Ventral gill raker count; 41, 42 and 43. Gill raker length (first and third ventral [1^st^ v. and 3^rd^ v.] and third dorsal [3^rd^ d.]).

To quantify phenotypic diversity, we measured 36 continuous morphometric traits and seven discrete meristic traits (Fig 3), all considered to be of ecological significance, of 576 estuarine smelt according to the procedures of Barrette et al. [28]. Jaw, head and eye measurements as well as gill arch dimensions and the number and length of gill rakers are of significance to foraging strategy and relative fin sizes and body dimensions are of significance to swimming dynamics.

### Data analysis

We applied a statistical procedure employing Principal Component Analysis, Discriminant Function Analysis and ANOVA models to quantify the nature, extent and sources of variation in phenotypic diversity. We first log transformed all morphometric data to approach multivariate normality. We then standardized morphometric measures for fish of different sizes as we are concerned principally with differences in ecologically relevant morphological traits independent of the size effect. We performed a Principal Components Analysis (PCA) [31, 32] with target populations pooled to generate factorial axes. We removed the size effect by regressing the value of each variable for each fish on the first factorial axis that is largely explained by variation in size among fish. The residual values obtained were thus used to analyze intraspecific morphological diversity [28]. The size effect revealed by the first factorial axis involves all morphometric measurements, not just body length. Although we removed the size effect, the data are not allometry free and axes may still remain partly correlated with body size measurements. Meristic measures were used without any transformation as they were independent of size in smelt. This approach also allowed us to combine morphometric and meristic traits within a common analytical framework.

We first tested if the morphology of mixed-ancestry populations varied as a function of the relative contribution of the two races using a multivariate regression model. Because the variables were not multivariate normally distributed, exact MANOVAs based on permutation tests were used. As the regression was highly significant (see below), we formed 2 groups of mixed populations, one dominated by the Acadian race (relative contribution of the Acadian race varying from 0.65 to 0.87, Acadian-mixed) and a second dominated by the Atlantic race (relative contribution of the Acadian race varying from 0.10 to 0.38; Atlantic-mixed, Table 2).

We then analyzed the two races (Acadian and Atlantic) occurring in populations characterized by the absence or presence of mixed ancestry (pure Acadian, pure Atlantic, Acadian-mixed, Atlantic-mixed) using a nearest neighbor nonparametric discriminant function analysis (DFA). We employed 3 contrasts to test several hypotheses. First, we hypothesized that the morphology of groups composed of populations derived from each historical race and showing no evidence of genetic mixture are divergent, as demonstrated in lacustrine populations by Barrette *et al*. [29]. Secondly, we compared the morphology of populations dominated by one founding race with that of populations derived from both founding races. *P*-values were calculated for pairwise comparison of the groups on the most significant DFA axes with a mixed ANOVA model using populations as a random effect. Corrections were applied to *P*-values to control the type I error rate using the Bonferroni method. We also used loadings on the different axes of the DFAs to identify phenotypic correlations associated with different groups. We identified significant loadings using Fisher’s inverse hyperbolic tangent transformation [33] with a Bonferroni correction to control the type I error rate at the 1% level. We also evaluated the success of reclassification using the leave-one-out cross-validation method [34].

We quantified, with random ANOVA models, the relative importance of the major among- and within-population sources of variation acting to differentiate groups according to the canonical axes. The random ANOVA model provides a quantitative means to partition the variation observed on each canonical axis among the designated groups. The variance components were estimated by the maximum likelihood method [35] via the procedure VARCOMP of SAS (version 9.3, SAS Institute Inc, NC). Tests of significance on these variance components were made using the bootstrap technique with 5000 bootstrap replicates of each population [36]. The ANOVA model also quantified the within population variation that served as the error term. The random ANOVA model was fitted on the main canonical axes of the DFA using the following variance components: (1) pure Acadian vs pure Atlantic; (2) Acadian-mixed vs Acadian-pure; (3) Atlantic- mixed vs Atlantic pure (4) the remaining among-population variation, nested within groups (df = 11). We used this variance component to evaluate the importance of unaccounted sources of variation in influencing morphology.

In the case of a significant effect of genetic mixture, median values of traits significantly correlated with the discriminant functions were compared for evidence of transgressive segregation. Medians and their variance were used as the morphological trait values were not normally distributed. Traits of mixed-ancestry populations were considered as being transgressive if their medians were significantly smaller or larger than the smallest and largest median trait values, respectively, of populations showing no evidence of mixed ancestry of historical lineages [37]. We restricted this analysis to traits that contributed significantly to differentiating population groups in DFA. To calculate trait medians, the morphometric variables were back-transformed to their original scale, but expressed around an average fish (averaged for all morphological traits of the 576 fish) to remove the size effect (S1 Protocol). We then tested the equality of trait medians among groups using the median one-way nonparametric ANOVA. The equality of medians was rejected at *P*< 0.0167 following the Bonferonni correction for multiple comparisons.

### Populations and patterns of genetic admixture

#### Study site

Smelt of different age classes were sampled at different seasons during 2011–2012 throughout the St. Lawrence River (SLR; Fig 2, Table 2). The SLR extends 1600 km from the outlet of Lake Ontario to the Atlantic Ocean, and comprises three fluvial lakes connected to lotic sections, a freshwater estuary, a brackish estuary, and a lower (marine) estuary flowing into the Gulf of St. Lawrence (Fig 2) [38]. We sampled the lotic section of the river in the vicinity of Montreal, the freshwater estuary, the brackish estuary, the maritime estuary (including the Saguenay fjord) and Chaleur Bay (Fig 2). The brackish estuary encompasses two ecologically distinct environments. A steep rocky shoreline and deep channel characterize the north shore and extensive shallow mud flats characterize the south shore of the estuary. These two environments are exploited by the two distinct sympatric (in the geographical sense) populations, the ‘north-shore’ and ‘south-shore’ populations (see above).

#### Sampling

Three categories of fish were sampled. Firstly, reproductive fish were sampled on known spawning grounds to examine the degree of reproductive isolation among putative local populations. Secondly, we sampled adult smelt captured during the winter recreational ice fishery and, thirdly, young-of-the-year (YOY) fish, at several sites (Fig 2, Table 2) so as to increase the probability of discovering previously unsampled populations in the St. Lawrence. The locations of spawning grounds of 2 putative local populations were unknown and thus represented by YOY fish; sites 1 and 3 (Fig 2, Table 2). The spawning grounds of the north-shore population are unknown, but the definition of this population is based on the capture of post-spawning adults in the vicinity of Île aux Coudres (site 5, Fig 2), on the north shore of the St. Lawrence estuary, prior to the spring spawning season of the south shore population [39]. A sample of these fish thus served to genetically characterize the north-shore population. Finally 2 lacustrine populations were sampled. Lake Champlain smelt are pure mtDNA Atlantic race and provided the opportunity to relate a pure Atlantic mtDNA population with a nuclear genomic profile. A sample of adult fish was obtained from Jacques Cartier Lake (Fig 2, Table 2), an isolated population of lacustrine smelt that was recently founded by transplanting fish from source populations that were representative of both historical races. Although not estuarine, this population served to validate our ability to detect evidence of genetic admixture of the founding races.

#### Genetic characterisation

We extracted DNA from EtOH-preserved tissues with the QIAGEN blood and tissues extraction kit (QIAGEN), and quantified DNA concentration by spectrophotometry. We generated AFLP fragments for 447 individuals following the AFLP Plant Mapping protocol (Applied Biosystems Inc. Foster City, CA, USA). We used ca. 100 ng of DNA for the restriction-ligation step and two *Eco*RI/*Mse*I primer pairs in selective PCRs (ATG/CAG and ACC/CAC). Fragments were migrated on ABI capillary sequencer and peaks with a minimum relative fluorescence of 100 units were scored manually using GeneMapper 3.7. For a subset of 153 individuals scored for AFLPs (Table 2), we sequenced a 565-bp segment of the ND5/ND6 mitochondrial DNA segment containing diagnostic differences between the Acadian and Atlantic mtDNA lineages [23].

We used structure 2.3.3 [40, 41, 42] to determine the number of distinct genetic clusters within the SLR (50,000 burn-in followed by 200,000 iterations, admix model, with and without prior information on sampling location (Loc Prior), 10 runs for each of *K =* 1–20). We used the criteria of Pritchard *et al*. [40] and Evanno *et al*. [43] to infer the most likely number of groups (*K*). Figs. were made with distruct [44]. The most likely number of clusters was considered to distinguish contemporary populations within the system. As a complement, pairwise F_ST_ between sample sites were estimated based on the distance matrix of pairwise differences between AFLP profiles with arlequin 3.5 [ 45 ]. Finally, an analysis of molecular variance (AMOVA) was conducted to assess if clusters account for a significant proportion of genetic variance beyond that occurring among samples within clusters. Calculations were done using 1000 permutations in arlequin 3.5. [45].

To assess the patterns of admixture between the two founding races, we considered the results of structure with *K* = 2. First, we assessed the extent of genome admixture of the founding nuclear races using two independent methods. Namely, we used *q*, the Bayesian coefficient of ancestry provided by structure for *K* = 2, and *h*, the maximum-likelihood hybrid index estimated with introgress [46, 47]. For the latter, based on the results of structure, we considered samples from Montreal and du Loup River as representative of the reference and alternative parental populations for nuclear DNA, respectively.

Finally, we tested the correspondence between the nuclear and mtDNA evidence for two ancestral races using a logistic regression analysis [48], where the binary response variable was assignment to either the Atlantic or Acadian mtDNA race. Specifically, we calculated the probability of being classified in the mtDNA Atlantic race as a function of the inferred ancestry (*q*) in cluster 2 (when *K* = 2) and, in a second model, as a function of *h*, the maximum likelihood hybrid index. The Hosmer-Lemeshow test was used to confirm the goodness of fit of both models to the data. To quantify the concordance between the nuclear and mtDNA races, the area under the receiver operating characteristics (ROC) curve was calculated.

## Results

### Populations and morphological diversity

**Fig 4:**
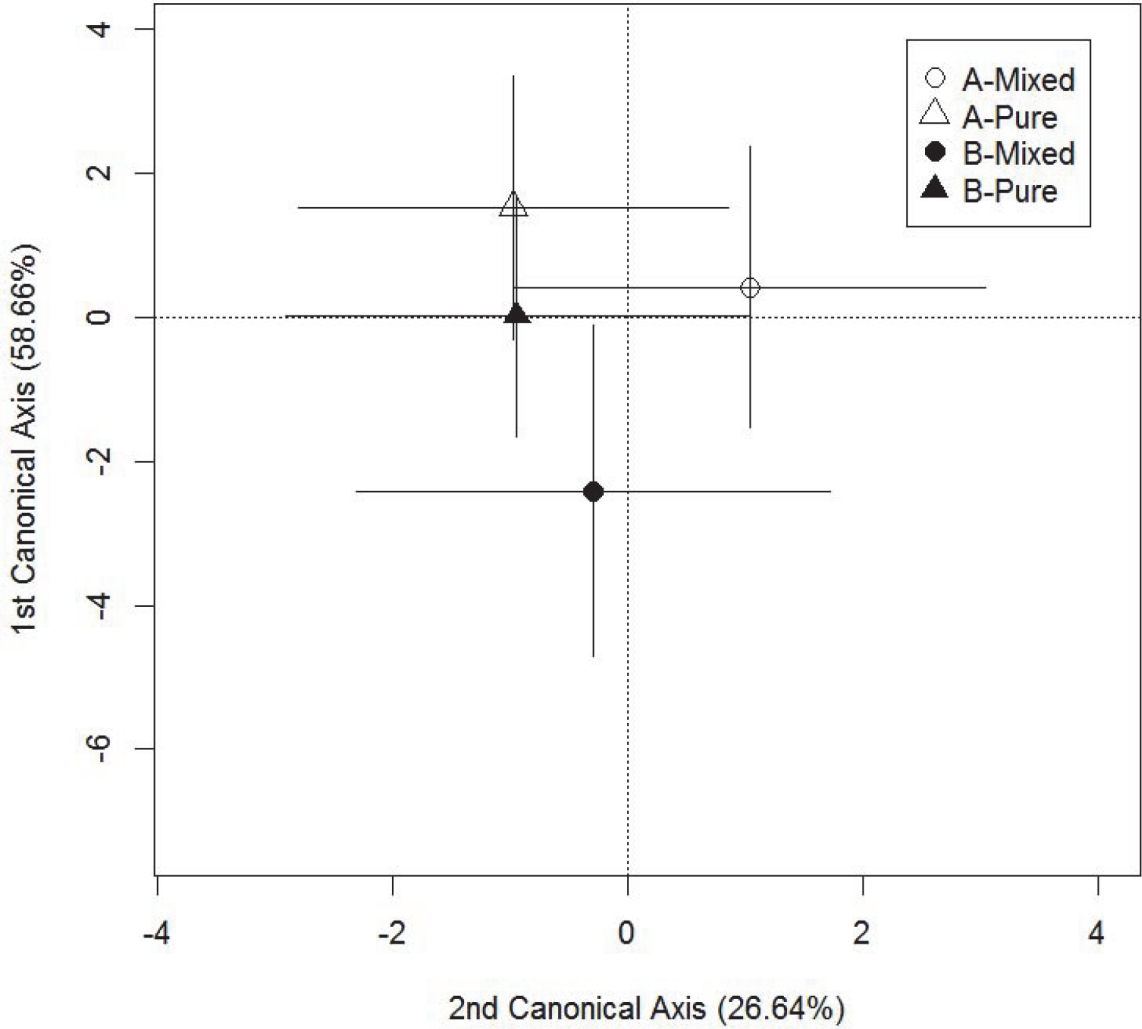
Discriminant function analysis significantly differentiating smelt from populations characterized by the absence or presence of genetic admixture of the two glacial races. Canonical axes have a relative discriminative power of 58.66% (axis 1; *P*< 0.0001) and 26.64% (axis 2; *P*< 0.0001); N = 576. For morphological traits that contribute significantly to the formation of each canonical axis, see table 4. Each point represents the mean group value. Horizontal and vertical bars show the 95% confidence intervals around the mean value. Open symbols denote groups from the Acadian race (A) and filled symbols denote groups from the Atlantic race (B). Triangles denote fish from populations with no evidence of genetic admixture of the two races (A-pure, B-pure) and circles denote groups of hybridized fish of predominantly Acadian (A-Mixed) or Atlantic (B-Mixed) origin.

The preponderance of the Atlantic mtDNA lineage in populations of the northern Gulf of St. Lawrence and Newfoundland (Fig 2) suggests a different dispersal history of the Atlantic race than previously surmised [22]. Secondary contact of the two races thus appears not to have been restricted to the St. Lawrence estuary. The morphology of the mixed-ancestry populations differed according to the relative contributions of the founding races. The multivariate regression model was highly significant (*F*_Wilk’s lambda_ = 21.43; df = 42, 314; *P*< 0.0001). When grouping estuarine smelt populations characterized by the extent of mixture of the two historical mtDNA races (pure Acadian, pure Atlantic, Acadian-mixed, Atlantic-mixed), the morphologies of the 4 groups differed significantly (MANOVA: *F*_Wilk’s lambda_ = 11.97; d.f. = 126, 1591.9; *P*< 0.0001). All three discriminant functions were significant (*P*< 0.0001), with the first function explaining 58.7% of the variance, and the second and the third functions explaining 26.6% and 14.7% respectively. Reclassification of smelt to their group of origin revealed that smelt from populations of mixed ancestry were more successfully reassigned (Acadian-mixed, 88.6%; Atlantic-mixed, 84.2%) than smelt from populations of pure ancestry (pure Acadian, 67.7.0%; pure Atlantic, 61.1%). The suite of correlated traits accounting for the greatest degree of morphological divergence discriminated smelt from Atlantic-mixed populations (Fig 4). The Atlantic-mixed populations differed significantly from all other groups on this axis (*P* = 0.0019). No significant differences occurred among the remaining 3 population groups. Contrasting Atlantic-mixed populations with pure Atlantic populations explained 47.8% of the variance associated with this function (Table 3). Contrasting pure-Atlantic and pure Acadian population groups explained 40.1% of the total variance (Table 3), reflecting the historical divergence of the two historical races (S1 File). The third contrast, comparing the pure Acadian populations with Acadian-mixed populations, explained less than 2% of the variation on this axis. Finally, the remaining among-population variation was only 2.4%. Twenty-two morphological traits were significantly correlated with the first axis (Table 4). Briefly, Atlantic-mixed populations were characterized by bigger eyes, longer gill arches, deeper heads, larger jaw dimensions and longer gill rakers relative to all other estuarine population groups.

**Table 3:** Variance explained by historical lineage and genetic admixture when smelt populations are subdivided in four groups in a DFA: estuarine smelt populations of pure Acadian and Atlantic origin (Ac pure and At pure), estuarine smelt populations showing evidence of admixture with the alternate historical lineage (Ac mixed, At mixed).

Axis 1	d.f.	Variance estimate	Bootstrap standard error	Wald chisquare	*P-*value	Part of the total variance
Ac_pure_ vs At_pure_	1	4.1830	0.3455	146.589	<0.0001	40.06%
Ac_mixed_ vs Ac_pure_	1	0.2004	0.0590	11.516	0.0007	1.92%
At_mixed_ vs At_pure_	1	4.9952	0.3382	218.187	<0.0001	47.84%
Pop_(group)_	11	0.2513	0.0463	29.399	<0.0001	2.41%
Error	561	0.8124	0.0546	221.302	<0.0001	7.78%
Total	575	10.4423				
**Axis 2**	**d.f.**	**Variance estimate**	**Bootstrap standard error**	**Wald chisquare**	***P*-value**	**Part of the total variance**
Ac_pure_ vs At_pure_	1	0.3049	0.0948	10.337	0.0013	10.83%
Ac_mixed_ vs Ac_pure_	1	1.4886	0.1576	89.25	<0.0001	52.90%
At_mixed_ vs At_pure_	1	0.0000	0.0048	0.000	1.0000	0.00%
Pop_(group)_	11	0.1343	0.0340	15.64	<0.0001	4.77%
Error	561	0.8864	0.0522	288.008	<0.0001	31.50%
Total	575	2.8142				

The random ANOVA models (with crossed and nested factors) partitioned variance, on the two main canonical axes (Axis 1 and Axis 2) of the DFA, among the following contrasts (1) Ac pure vs At pure, (2) Ac mixed vs Ac pure, (3) At mixed vs At pure, (4) the remaining among populations variability nested within groups “Pop_(group)_”, and (5) the within population variability “Error”. Axis 1- the first discriminant function. Axis 2- the second discriminant function.

**Table 4:** Highly-correlated morphological traits, composing the two main axes of variation of a DFA differentiating rainbow smelt grouped according to glacial race and genetic admixture.

**Axis 1**		**Axis 2**	
Variables	Loadings	Variables	Loadings
**Eye to maxillary l.**	-0.4037	**Maxillary depth**	- 0.2583
**Ventral gill arch l.**	-0.3931	**Anal fin base**	- 0.2479
**Head depth**	-0.3604	**Caudal peduncle d**	- 0.2476
**Lower jaw l.**	-0.3496	Gill raker (3rd ventral) l	- 0.2008
**Upper jaw l.**	-0.3433	Anal fin l.	- 0.1909
**Maxillary l.**	-0.3390	Caudal fin l.	- 0.1739
Eye area	-0.2403	**Dorsal gill arch l.**	0.1865
Eye diameter	-0.2370	Dorsal fin to fork l.	0.2139
**Dorsal gill arch l.**	-0.2150	Fork to anal fin l.	0.2143
**Snout l.**	-0.1640	**Post-orbital l.**	0.2162
Gill raker l. (1st ventral)	-0.1531	**Ventral gill arch l.**	0.2421
**Fork to anal fin l.**	0.1708	Fork length.	0.2827
**Trunk l.**	0.1995	**Trunk l.**	0.2896
Dorsal fin l.	0.2306	**Anal fin to dorsal fin l.**	0.3382
**Caudal peduncle d.**	0.2396	**Head width**	0.4183
Anal fin to dorsal fin l.	0.2544		
Pelvic fin base	0.2674		
Pectoral fin base	0.2764		
**Anal fin base**	0.3132		
**Dorsal fin base**	0.3580		
**Dorsal fin to fork l.**	0.3883		
**Fork l.**	0.3897		

Significant loadings were greater than +0.152 or lower than -0.152 at the 1% level (Bonferroni correction, alpha = 0.01/(2 x 42) = 0.000119); Fisher’s inverse hyperbolic tangent transformation. Traits with a negative loading on DFA axis 1 have higher trait values for mixed- Atlantic populations (located at the negative end of axis 1) and lower trait values for all other groups (located at the positive end of axis 1). Conversely, a positive loading on this axis had a lower trait value for mixed-Atlantic populations. Traits with a positive loading on axis 2 have higher trait values for mixed-Acadian populations and lower trait values for all other groups. Conversely, a negative loading on this axis had a lower trait value for mixed- Acadian populations. l.- length, d.- depth, ct.- count. Traits in bold print signify transgressive traits (see S1 Table for values).

The second suite of correlated traits (function 2), discriminated smelt from Acadian-mixed populations (Fig 4). This population group differed significantly from all other population groups on this axis (*P* = 0.008) whereas no significant differences occurred among the remaining 3 groups. Contrasting Acadian-mixed populations with pure Acadian populations explained 52.9% of the variance associated with this function (Table 3). Contrasting groups with no evidence of mixed ancestry (pure Acadian, pure Atlantic) explained 10.8% of the total variance. The third contrast, comparing pure Atlantic race populations and Atlantic-mixed populations explained no variance on this function. The remaining among-population variation, nested within groups, accounted for less than 5% of total variation. Fifteen morphological traits were significantly correlated with the second axis (Table 4). Briefly, Acadian-mixed populations were characterized by wider heads, longer body dimensions, shorter gill rakers and smaller fins.

Of the 22 morphological traits contributing significantly to the first discriminant axis and the 15 contributing to the second axis, 15 and 9, respectively, exhibited median trait values that were either significantly smaller or larger in mixed ancestry populations when compared with populations with no evidence of mixed ancestry (Table 4, S1 Table).

### Populations and genetic admixture

Among the 447 smelt collected in the SLR and successfully analyzed, a total of 108 AFLP bands were unambiguously scored, of which 64 were polymorphic. However, 35 of these polymorphisms were uninformative, occurring at a frequency lower than 5% in any one sample. We thus retained 29 bands for subsequent analyses. Although only 29 bands were retained, we considered this adequate for population definition. The associated F_ST_ values were highly significant (see S2 Table) and the genotyping error rate extremely low (1.9%; [49]). Furthermore, the AMOVA conducted among the 7 genetic groups defined in structure (see below) explained a large proportion of the total genetic variance (14.9%, *P*< 0.00001), almost 5 times more than that accounted for by variance among samples within groups (3.2%, *P*< 0.00001).

**Fig 5:**
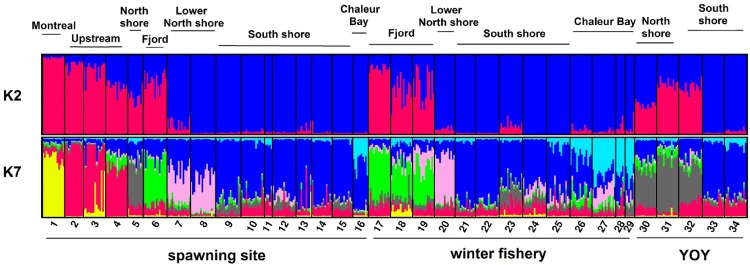
Estimated population structure as obtained in structure with Loc Prior. Each individual is represented by a thin vertical line, which is partitioned into *K* colored segments that represent the individual's estimated ancestry within each *K* cluster. Two values of *K* are presented; upper panel: *K* = 2 (optimal *K* using Evanno’s criterion) and lower panel: *K* = 7 (optimal K using Pritchard’s criterion. Black lines separate individuals of different sampling sites. Population clusters are labeled above the Fig., individual sampling sites and sample category, below the Fig.

The cluster analysis performed with structure using Pritchard’s criterion supported the definition of *K* = 7 distinct genetic clusters along the SLR (Fig 5). These include 2 populations located at the upstream end of the SLR (table 2; Population 1 sampled at site 1 (yellow in Fig 2 and 5), and Population 2 sampled at sites 2, 3 and 4 (red in Fig 2 and 5)), the North Shore Population sampled at site 5 (grey in Fig 2 and 5), the Fjord Population sampled at site 6 (green in Fig 2 and 5), the Lower North Shore Population at sites 7 and 8 (pink in Fig 2 and 5), the South Shore Population at sites 10, 11, 12, 13, 14, 15 and 9 (dark blue in Fig 2 and 5) and the Chaleur Bay Population at site 16 (light blue in Fig 2 and 5). There are marked differences in the spatial extent of clusters, with the South Shore Population forming a large, undifferentiated group along the estuary’s south shore (with an extension to the north shore (site 9)) while several distinct clusters are found along the estuary’s north shore. The distribution of the 7 groups is similar among the winter fishery samples (samples17-29) and among the YOY samples (samples 30–34) (Fig 2 and 5), indicating that the distribution of these life stages reflects that of the local spawning populations. Pairwise F_st_ values between samples representative of 16 known or presumed local spawning groups confirmed this pattern. Of 120 pairwise comparisons, only 22 were not significant (S2 Table); 19 of these involved spawning sites of the South Shore Population.

The same cluster analysis using Evanno’s criterion supported the definition of *K* = 2 distinct genetic clusters (Fig 5). These clusters exhibit a clear gradient along the upstream-downstream and the north shore-south shore axes (Fig 5). Cluster 1 (blue in Fig 5) dominates samples located along the south shore (samples 10–15), downstream on the lower north shore (samples 7–9) and in Chaleur Bay (sample 16) (Fig 5). In contrast, cluster 2 (red in Fig 5) is largely restricted to the upstream sites and the north shore of the SLR among spawning samples (site 1–6). Once again, the distribution of the 2 groups is similar among the winter fishery samples and among the YOY samples.

**Fig 6:**
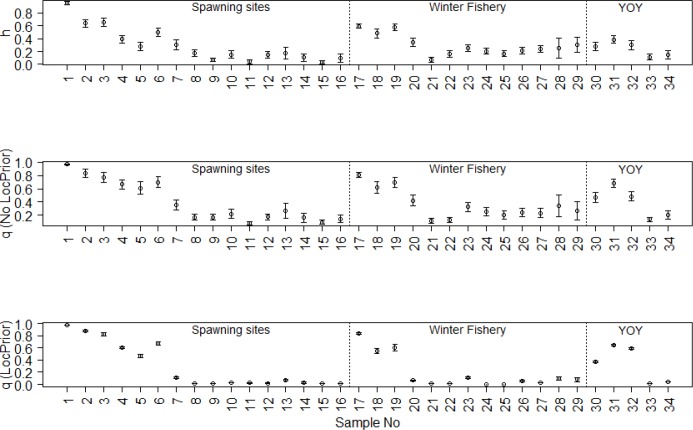
Sample mean and S.E.M of (a) Hybrid index (*h*) and Bayesian admixture proportions (*q*), with (b) and without (c) Loc Prior. The 34 samples are arranged as per Fig 3 in downstream order with spawning sites (1–16) followed by winter fishery samples (18–29) and the YOY samples (30–34).

Estimates of the degree of genetic admixture between the two races (*K* = 2) indicate that admixture occurred principally upstream and along the north shore of the SLR with little evidence of admixture occurring along the south shore (Fig 5). The hybrid index (*h*) and the coefficient of ancestry (*q*, calculated without Loc Prior) (Fig 6) decline downstream from Montreal, showing considerable admixture in the populations of Lake Jacques Cartier, the North Shore Population and the Fjord Population. Values of *h* and *q* remain low in the South Shore, Chaleur Bay and Lower North Shore populations. This gradient is repeated in all three sample types. This tendency is most evident when calculating *q* with Loc Prior (Fig 6). Loc Prior tends to polarize *q* values, underestimating *q* when the hybrid index approaches 0 and overestimating *q* when the hybrid index approaches 1 thus amplifying the distinction of genetically admixed populations. The population from Lake Jacques Cartier (site 4) conforms to the expectation of a genetically admixed population (as it was so stocked). Similarly, the North Shore Population (spawners, 5 and YOY, 30, 31 and 32), and the Fjord Population (spawners, 6 and winter fishery, 17, 18 and 19) are also admixed relative to all other populations.

Finally, the logistic regression models revealed that the probability of being classified in the mtDNA Atlantic race increases significantly with *q* or *h* (respectively, Wald chi-square = 19.27, df = 1, *P*<0.0001; Wald chi-square = 12.34, df = 1, *P* = 0.0004). In the first and second models, respectively, 70.9% and 67.7% of the predicted probabilities were in concordance with observed assignments. Moreover, variables *q* and *h* are highly correlated to each other (Pearson correlation *r* = 0.89) and the inferred ancestry (*q*) is the most important of the two variables for classifying individuals. After considering the effect of *q*, the index *h* explains no further part of variation (Wald chi-square = 1.25, df = 1, *P* = 0.2628). The blue cluster illustrated in Fig 5 thus corresponds to the Acadian mtDNA race and the red cluster illustrated in Fig 5 corresponds to the Atlantic mtDNA race.

## Discussion

The morphology of mixed-ancestry populations (co-occurrence of Acadian and Atlantic mtDNA races) varied as a function of the relative contribution of the two races to estuarine populations, supporting the hypothesis of genetic admixture as opposed to ancestral polymorphism. If the lineage composition of these populations were uniquely due to the retention of ancestral polymorphisms, there would be no reason to expect that populations dominated by one or the other lineage would be consistently morphologically distinct relative to populations comprising a single lineage. Pure estuarine populations of the two races are morphologically distinct, as observed in lacustrine populations [29]. We observed that populations comprising both ancestral mtDNA lineages did not exhibit intermediate morphologies relative to populations comprising a single lineage. On the contrary, the genetic mixture of the ancestral mtDNA races generated suites of correlated morphological traits, many of which exceeded the parental trait values. The morphology of Atlantic-mixed populations explained the greatest part of the total morphological variation revealed by our analyses, representing the major source of variation on the first discriminant axis. Furthermore, over 68% of morphological traits distinguishing Atlantic-mixed populations exhibited extreme values relative to pure Atlantic populations, consistent with the hypothesis of transgressive segregation. The morphology of Acadian-mixed populations also differed significantly from all others on the second axis. However, as the second axis explained less than half of the variance accounted for by the first axis, contrasting Acadian-mixed populations with pure Acadian populations accounts for only approximately one half of the variance explained by contrasting Atlantic-mixed populations with pure Atlantic populations. Sixty percent of traits distinguishing Acadian-mixed populations exhibited extreme values relative to pure Acadian populations.

The observations presented above are consistent with the hypothesis of transgressive segregation. Evidence for genetic admixture at the level of the nuclear gene pool, however, provides only partial support for this hypothesis. The nuclear genome of rainbow smelt revealed clear evidence that two races were admixed within some populations of the purported secondary contact zone within the St. Lawrence system. In particular, the admixture of the two nuclear races is pronounced in mixed-ancestry populations dominated by the Atlantic race, the same populations showing the greatest degree of morphological differentiation. Earlier studies of the St. Lawrence sympatric smelt populations identified head length, eye area and jaw length as being responsible for the strong morphological differentiation between the ‘north-shore’ and ‘south-shore’ St. Lawrence estuary populations [27, 28]. Relative to smelt populations of the pure Atlantic race, populations of admixed smelt of Atlantic origin also exhibit significantly bigger head and jaw dimensions (including a longer upper and lower jaw and a bigger eye diameter, head depth and snout length) than all other estuarine populations, the same traits that were previously associated uniquely with the ‘north-shore’ population of the St. Lawrence estuary. Large jaws and big eyes are thus characteristic of at least 3 Atlantic-mixed populations found throughout the St. Lawrence system.

Coincidentally, many of the populations comprising the St. Lawrence smelt complex reside in the fluvial estuary, along the north shore of the middle estuary and in the Saguenay fjord, areas associated with the greatest degree of admixture of the 2 nuclear gene pools. This may be largely due to the strong physical heterogeneity of the region, favoring reproductive isolation through philopatry. These areas and the south shore of the estuary are only separated by several tens of kilometers, well within the dispersal capacity of smelt [19]. Atlantic-mixed smelt may be at a competitive advantage in the more heterogeneous estuarine environments because of greater niche-partitioning opportunities associated with large gape size and better vision. Differences in mouth morphology have been implicated as a major determinant of variation in prey types and sizes consumed by predatory fishes [50,51]. Large-gape predators feed on small prey sizes while also being able to feed on large prey that are unavailable to smaller predators. In addition, as they grow, they simply incorporate larger prey as their increased gape size permits [52]. Among many marine species, gape size is a good indicator of maximum prey size consumed [51] and trophic level [53]. In addition to the benefits of large gape size, big eyes facilitate foraging on elusive prey in mid-water [54]. We speculate that the diversification in craniofacial phenotypes documented here for admixed smelt populations may be key to adaptive radiation, as hypothesized for vertebrates in general [55].

The pattern of admixture based on nuclear DNA did not always reflect the pattern of admixture suggested by mtDNA. The Atlantic mtDNA lineage composed approximately 20% of the mtDNA gene pool of the South Shore and Chaleur Bay populations, but these populations showed little evidence of introgression of the two nuclear gene pools and differed morphologically from pure Acadian populations to a far lesser degree. As a result, the correspondence between nuclear and mtDNA races, albeit significant, is not perfect. Although we cannot discount completely the retention of ancestral polymorphism as a contributing factor, the lack of complete concordance between nuclear and mtDNA races most likely reflects introgression of mtDNA lineages between the two ancestral nuclear DNA races.

The lack of evidence for admixture of the two nuclear DNA races along the south shore, in spite of evidence for the likely introgression of the mtDNA races in this area, may involve active selection against the Atlantic genome. In contrast to the north shore of the SLR, the smelt sampled in different spawning tributaries along the south shore of the SLR shows little evidence of introgression of the nuclear genome and no contemporary population genetic structure. Furthermore, the Acadian-mixed populations occupying the St. Lawrence and the estuaries of New Brunswick exhibit little morphological diversification relative to populations comprising uniquely the Acadian lineage. As selection is typically expected to favor locally adapted genotypes and can act against admixed individuals, there may be some conditions under which admixture will have negative impacts on population fitness [13]. Although speculative, the Acadian race phenotype may be well suited to the shallow, shoal environment typical of the south shore of the estuary. Admixture with the Atlantic morphotype may have contributed to the loss of advantageous parental traits with negative impacts on fitness. Another possibility is that differential phenotypic plasticity between the two ancestral races may have played a role, as has been proposed for postglacial populations of threespine stickleback (*Gasterosteus aculeatus*) that evolved under greater seasonal temperature extremes following invasion of lakes from the sea [56]. If we speculate that the Atlantic race possesses greater phenotypic plasticity than the Acadian race, such plasticity may have been disadvantageous in the relatively homogenous shallow, coastal environments occupied by the Acadian race resulting in selection against admixed individuals. We are unable to test this hypothesis.

Our findings are not entirely consistent with the biogeographical hypothesis of two routes of postglacial re-colonization of North-east North America by smelt [22]. The presence in Newfoundland (located next to the putative Acadian refugium) of two populations comprising a mtDNA lineage diagnostic of the Atlantic race (sites n, o; Fig 2) and the presence of a mixed ancestry population on the north coast of the Gulf of St. Lawrence dominated by the Atlantic race (site k; Fig 2) suggests that post-glacial dispersal of the Atlantic race may have been more widespread than the single inland route proposed by Bernatchez [22]. The observation of significant genetic and morphological discontinuities in estuarine populations of smelt in Newfoundland and the Canadian Maritime provinces has also led to the questioning of colonization of these waters uniquely from the Acadian refugium [57]. If the Atlantic race also dispersed via a maritime route rather than a unique inland route, admixture of the Atlantic and Acadian races may have occurred at numerous locations outside the St. Lawrence estuary. The observation, on the north coast of the Gulf of St. Lawrence, of a mixed-ancestry population dominated by the Atlantic race exhibiting significantly bigger head and jaw dimensions tends to support this possibility. However, far more extensive genetic and morphological analyses of the Gulf of St. Lawrence and Newfoundland smelt populations are needed to re-evaluate the biogeographical history of the species.

We set out to find evidence that genetic admixture within rainbow smelt populations contributed to the morphological diversification and population structure of the species in northeastern North America rather than to a net loss of diversity through genetic homogenization and an averaging of morphological features. We have presented two contrasting outcomes for the role of natural selection within the smelt population complex, acting both for and against admixed genomes. Other evolutionary processes, not documented in this study, are no doubt involved in the evolution of this species complex. Nevertheless, the presence of extreme phenotypes associated with admixed populations of Atlantic origin implicates transgressive segregation in the diversification of the estuarine population complex. It has been proposed that genetic admixture among the cichlid fishes of the East African rift lakes may increase evolvability by providing new phenotypic variation previously unseen by selection, through transgressive segregation [58, 59]. Historical events involving postglacial contacts between closely related lineages have also been hypothesized to contribute to the extreme phenotypic variability of North American ciscoes *Coregonus* spp. [60]. The hypothesis that organisms from disparate native-range sources form genetically admixed populations with elevated genetic variation that may facilitate invasion success [11, 61] may serve as a present-day analogy of the dynamics of post-glacial colonization of new environments by previously isolated intraspecific races.

## Supporting Information

S1 Protocol Back transformation of measurements to an average fish. (DOCX)

S1 File The morphological distinction of saltwater and freshwater forms of rainbow smelt. (DOCX)

S1 Table Median values of morphological traits correlated with the first and second axes of the discriminant function analysis. (DOCX)

S2 Table F_ST_ pairwise comparisons. (DOCX)
