# Supplementary material for: Intraspecific Genetic Admixture and the Morphological Diversification of an Estuarine Fish Population Complex
Source: PLoS One. 2015 Apr 9;10(4):e0123172. doi: 10.1371/journal.pone.0123172 (PMC4391849; doi:10.1371/journal.pone.0123172)
Supplement: S1 Protocol — (DOCX) [file pone.0123172.s001.docx]

**S1-protocol. Supplementary Information**

**Back-transformation of measurements to an average fish**

To illustrate the differences among groups, the morphometric variables were back-transformed to their original scale, but expressed around an average fish to remove the size effect. For any variable, this back-transformation was done by first adding the mean of the log-transformed variable to their associated residual values, which were obtained by regressing this log-transformed variable on the first principal component axis. The next step consists of taking the exponential of this latter quantity, in order to express the variable on its original scale, and by multiplying the result by an appropriate constant in order to correct the bias. In other words, the constant was chosen to coincide the mean of the original variable with the mean of the back-transformed variable. In mathematical terms, the back-transformation value for variable j on fish i, $Y_{ij}^{(B)}$, was simply computed as followed:

$Y_{ij}^{(B)}=\exp\left(R_{ij}+\overline{\log Y_{.j}}\right)\times Cte$,

where

- $R_{ij}$is the residual of the regression of $\log(Y_{ij})$on the first principal component axis,
- $Y_{ij}$is the original value of variable j taken on fish i, i=1,…, n,
- $\overline{\log(Y_{.j})}=\sum_{i=1}^{n} \log(Y_{ij})/n$,
- $Cte=\left(\frac{\sum_{i=1}^{n} Y_{ij}}{\sum_{i=1}^{n} \exp\left(R_{ij}+\overline{\log Y_{.j}}\right)}\right)$ is the constant for the bias correction factor.
