# Supplementary material for: Intraspecific Genetic Admixture and the Morphological Diversification of an Estuarine Fish Population Complex
Source: PLoS One. 2015 Apr 9;10(4):e0123172. doi: 10.1371/journal.pone.0123172 (PMC4391849; doi:10.1371/journal.pone.0123172)
Supplement: S1 File — (DOCX) [file pone.0123172.s002.docx]

**S1-File. Supplementary information**

**The morphological distinction of saltwater and freshwater forms of rainbow smelt**.

Are there significant morphological differences between freshwater and saltwater forms of rainbow smelt and do the two ancestral races found in estuarine populations exhibit the same morphological divergence as observed in the lacustrine environment by Barrette et al (29)? To answer these questions, we grouped the 5 purebred estuarine populations (derived uniquely from the Acadian and Atlantic races, Table 2) and the 13 purebred freshwater populations from (29) according to historical race and environmental setting. Following MANOVA, we conducted a DFA to test the null hypothesis that the morphologies of the two historical races do not differ significantly according to environmental setting (lakes and estuaries). A random ANOVA model with nested and crossed factors was then fitted on the two main canonical axis of the DFA. The following variance components of the overall among-group variance were investigated: (1) mtDNA historical lineage (A (Acadian) or B (Atlantic): two levels); (2) environment (freshwater or estuarine: two levels); (3) variation due to the interaction of historical lineage and environment; and (4) the remaining among-population variation, nested within lineage and environment (18 levels). The null hypothesis predicts that there should be no interaction of historical race and environment and no significant major effect of environment.

When grouping rainbow smelt according to their historical race (Acadain and Atlantic) and their environment of origin (lakes and estuaries), the morphologies of the 4 groups differed significantly (MANOVA: *F* _Wilk’s lambda_= 23.63; d.f.= 129, 1933.6; *P*< 0.0001). Ten morphological traits were excluded from further analysis as they did not contribute significantly to distinguishing among groups. All three discriminant functions were significant (*P*< 0.0001), with the first function explaining 60.55% of the variance, function 2, 25.66% and function 3, an additional 13.79%. Reclassification of smelt to their group of origin was highly successful in all cases (leave-one-out cross-validation method; estuarine Acadian smelt (Race A) 100.0%; lake Acadian smelt 98.36%; estuarine Atlantic smelt (Race B) 98.95%; lake Atlantic smelt 97.2%). The suite of correlated traits accounting for the greatest degree of morphological divergence discriminated fish inhabiting the 2 environments (Figure S2), explaining 69.64% of the total variance associated with this discriminant function (Table S2-Axis 1). There was no significant contribution of historical lineage and no significant interaction between the two sources of variance (Table 2-Axis 1) on this axis.

| Axis 1 | d.f. | Variance estimate | Bootstrap standard error | Wald chisquare | P-value | Part of the total variance |
| --- | --- | --- | --- | --- | --- | --- |
| H | 1 | 0.165 | 0.069 | 5.72 | 0.0168 | 3.91 % |
| E | 1 | 2.939 | 0.138 | 456.49 | <0.0001 | 69.64 % |
| HxE | 1 | 0.083 | 0.060 | 1.93 | 0.1649 | 1.96 % |
| Pop_(HxE)_ | 14 | 0.205 | 0.037 | 30.47 | <0.0001 | 4.85 % |
| Error | 673 | 0.829 | 0.042 | 390.25 | <0.0001 | 19.65 % |
| Total | 690 | 4.220 |  |  |  |  |
| Axis 2 | d.f. | Variance estimate | Bootstrap standard error | Wald chisquare | P-value | Part of the total variance |
| H | 1 | 1.262 | 0.086 | 216.81 | <0.0001 | 48.50% |
| E | 1 | 0.323 | 0.052 | 38.45 | < 0.0001 | 12.41% |
| HxE | 1 | 0 | 0.006 | 0 | 1 | 0% |
| Pop_(HxE)_ | 14 | 0.120 | 0.031 | 15.09 | 0.0001 | 4.59% |
| Error | 673 | 0.897 | 0.051 | 315.47 | < 0.0001 | 34.50% |
| Total | 690 | 2.601 |  |  |  |  |

Table S2. Variance explained by historical race and environment when smelt populations are subdivided in four groups in a DFA: Acadian and Atlantic lacustrine smelt and Acadian and Atlantic estuarine smelt. The random ANOVA models (with crossed and nested factors) partitioned variance, on the two main canonical axes (Axis 1 and Axis 2) of the DFA, among (1) historical race “H”, (2) environment “E”, (3) the interaction of historical race and environment “HxE” , (4) the remaining among-populations variability nested within race and environment “Pop_(HxE)_”, and (5) the within population variability “Error”.


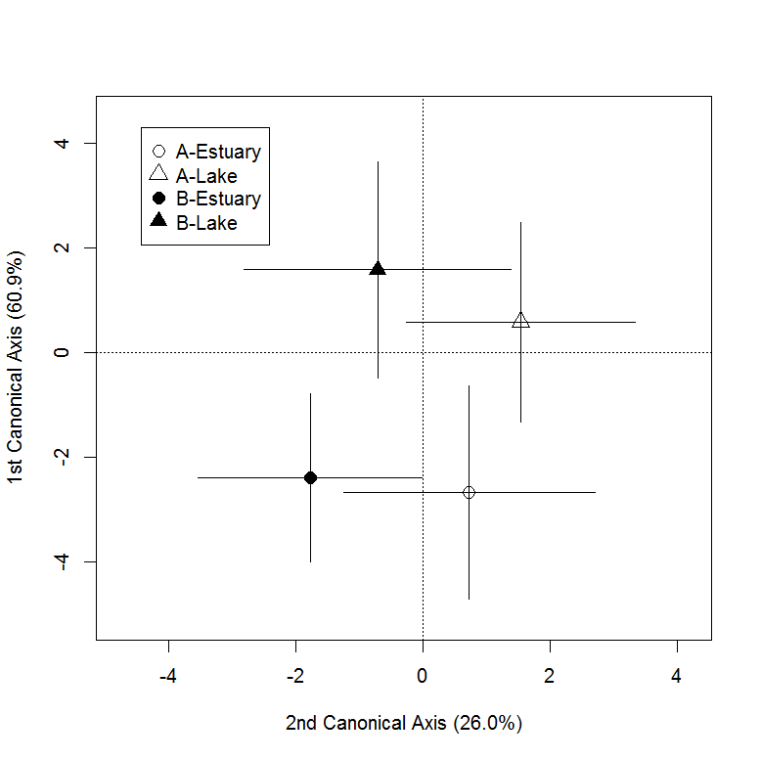


Figure S2. Discriminant function analysis significantly differentiating estuarine and lacustrine rainbow smelt derived from two historical races. Canonical axes have a relative discriminative power of 60.91% (axis 1; *P*< 0.0001) and 26.04% (axis 2; *P*< 0.0001); N= 691. Each point represents the mean group value. Horizontal and vertical bars show the 95% confidence intervals around the mean value. Circles denote groups of estuarine fish and triangles denote groups of freshwater fish. Filled symbols denote groups from the Atlantic historical race (clade B) and open symbols denote groups from the Acadian historical race (clade A).

The second most important suite of correlated traits discriminated fish differing in historical race (Fig. S2-Axis 2), explaining 48.50% of the total variance associated with this discriminant function . Environment explained a significant but less important part of the total variance of this axis (12.41%). There was no significant interaction between the two sources of variance (Table S2-Axis 2).

In conclusion, there was a major effect of environment and historical race. The two effects however were manifest on two independent (orthogonal) discriminant functions involving different suites of correlated morphological traits. The absence of any significant interaction between race and environmental setting on either of the two major axes indicates that the historical distinctiveness of the two races is largely retained regardless of environmental setting.
