# Supplementary material for: Intraspecific Genetic Admixture and the Morphological Diversification of an Estuarine Fish Population Complex
Source: PLoS One. 2015 Apr 9;10(4):e0123172. doi: 10.1371/journal.pone.0123172 (PMC4391849; doi:10.1371/journal.pone.0123172)
Supplement: S1 Table — (DOCX) [file pone.0123172.s003.docx]

**S1- Table. Supplementary information.**

**Table S1. Median values of morphological traits correlated with the first and second axes of the discriminant function analysis.**

| Variables (axis 1) | Atlantic mixed | Atlantic pure | Acadian pure |
| --- | --- | --- | --- |
| (-) **Eye to maxillary l.** | 9.5 (0.5)^a^ | 9.1 (0.4)^b^ | 8.8 (0.4)^c^ |
| (-) **Ventral gill arch l.** | 15.8 (0.6)^a^ | 15.0 (0.4)^b^ | 15.0 (0.4)^b^ |
| (-) **Head depth** | 15.0 (0.7)^a^ | 14.5 (0.9)^b^ | 14.2 (0.4)^c^ |
| (-) **Lower jaw l.** | 16.8 (0.8)^a^ | 16.2 (0.8)^b^ | 15.8 (0.5)^c^ |
| (-) **Upper jaw l.** | 16.4 (0.7)^a^ | 15.9 (0.7)^b^ | 15.5 (0.5)^c^ |
| **(-) Maxillary l.** | 11.7 (0.5)^a^ | 11.6 (0.4)^b^ | 11.2 (0.4)^c^ |
| (-) Eye area | 41.0(2.7)^a^ | 40.2 (2.5)^a^ | 37.9 (3.7)^b^ |
| (-) Eye diameter | 7.2 (0.3)^a^ | 7.1 (0.3)^a^ | 6.9 (0.3)^b^ |
| **(-) Dorsal gill arch l.** | 7.9 (0.4)^a^ | 7.6 (0.3)^b^ | 7.6 (0.3)^b^ |
| **(**-**)** S**nout l.** | 8.4 (0.4)^a^ | 8.3 (0.5)^b^ | 8.3 (0.3)^b^ |
| (-) Gill raker l. (1st ventral) | 4.7 (0.3)^a^ | 4.7 (0.3)^a^ | 4.5 (0.2)^b^ |
| **(+) Fork to anal fin l.** | 29.4 (1.5)^a^ | 29.9 (1.4)^b^ | 30.2 (1.4)^b^ |
| **(+) Trunk l.** | 44.0 (2.7)^a^ | 44.8 (1.7)^b^ | 44.9 (2.6)^b^ |
| (+) Dorsal fin l. | 21.5(1.2)^a^ | 21.9 (1.0)^a^ | 22.6 (0.9)^b^ |
| (+) **Caudal peduncle d.** | 8.6 (0.5)^a^ | 8.9 (0.5)^b^ | 9.1 (0.4)^c^ |
| (+) Anal fin to dorsal fin l. | 28.5 (1.7)^a^ | 28.9 (1.2)^a^ | 29.5 (1.6)^b^ |
| **(+)** Pelvic fin base | 4.6 (0.3)^a^ | 4.7 (0.3)^a^ | 5.0 (0.4)^b^ |
| (+) Pectoral fin base | 5.6 (0.4)^a^ | 5.7 (0.3)^a^ | 6.0 (0.3)^b^ |
| (+) **Anal fin base** | 20.5 (1.4)^a^ | 22.0 (1.1)^b^ | 22.2 (1.2)^b^ |
| **(+) Dorsal fin base** | 13.3 (0.9)^a^ | 13.7 (0.7)^b^ | 14.8 (0.9)^c^ |
| **(+) Dorsal fin to fork l.** | 70.0 (2.6)^a^ | 72.0 (2.0)^b^ | 73.3 (2.5)^c^ |
| **(+) Fork length** | 156.4 (4.5)^a^ | 158.4 (3.0)^b^ | 161.4 (4.0)^c^ |

| Variables (axis 2) | Acadian mixed | Acadian pure | Atlantic pure |
| --- | --- | --- | --- |
| (-) **Maxillary depth** | 2.6 (0.2)^a^ | 2.7 (0.2)^b^ | 2.7 (0.2)^b^ |
| (-) **Anal fin base** | 21.4 (1.3)^a^ | 22.2 (1.2)^b^ | 22.0 (1.1)^b^ |
| **(-) Caudal peduncle d.** | 8.8 (0.5)^a^ | 9.1 (0.3)^b^ | 8.9 (0.4)^a^ |
| (-) Gill raker (3rd ventral) l. | 4.7 (0.3)^a^ | 4.7 (0.3)^a^ | 4.9 (0.3)^b^ |
| (-) Anal fin l. | 12.1 (0.8)^a^ | 12.3 (0.7)^b^ | 12.2 (0.8)^ab^ |
| (-) Caudal fin l. | 25.1 (0.9)^a^ | 25.3 (1.0)^a^ | 25.8 (1.0)^b^ |
| **(+) Dorsal gill arch l.** | 7.8 (0.3)^a^ | 7.6 (0.3)^b^ | 7.6 (0.3)^b^ |
| (+) Dorsal fin to fork l. | 73.2 (2.0)^a^ | 73.3 (2.5)^a^ | 72.0 (2.0)^b^ |
| (+) Fork to anal length | 30.4 (1.2)^a^ | 30.2 (1.4)^ab^ | 29.9 (1.4)^b^ |
| **(+) Post-orbital l.** | 17.5 (0.8)^a^ | 17.1 (0.8)^b^ | 17.0 (0.7)^b^ |
| **(+) Ventral gill arch l.** | 15.4 (0.6)^a^ | 15.1 (0.5)^b^ | 15.1 (0.5)^b^ |
| (+) Fork length. | 161.7 (4.1)^a^ | 161.4 (4.0)^a^ | 158.4 (3.0)^b^ |
| (+) **Trunk l.** | 45.9(2.6)^a^ | 44.9 (2.6)^b^ | 44.8 (1.7)^b^ |
| (+) **Anal fin to dorsal fin l.** | 30.2 (1.6)^a^ | 29.5 (1.6)^b^ | 28.9 (1.3)^c^ |
| (+) **Head width** | 15.6 (1.0)^a^ | 14.7 (0.9)^b^ | 14.8 (1.1)^b^ |

S1 TABLE. The median values (mm) of the morphological traits correlated significantly with the first axis (upper section) and the second axis (lower section) of the DFA that significantly differentiated smelt from populations characterized by the absence (Acadian pure (n= 124), Atlantic pure (n= 95)) or presence (Atlantic mixed (n= 120), Acadian mixed (n= 198)) of genetic admixture of the two historical races. Gini’s mean difference was used as a robust estimator of the standard deviation (between parentheses). Different upper-case letters indicate significantly different medians (*P*< 0.0167). Traits in bold print signify transgressive traits; the median trait value of hybridized populations (Atlantic mixed, axis 1, and Acadian mixed, axis 2) are significantly smaller or greater than the median trait value of purebred populations (Atlantic pure, Acadian pure). (-) traits negatively correlated with the discriminant axis, (+) traits positively correlated with the discriminant axis.
