# Supplementary material for: Intraspecific Genetic Admixture and the Morphological Diversification of an Estuarine Fish Population Complex
Source: PLoS One. 2015 Apr 9;10(4):e0123172. doi: 10.1371/journal.pone.0123172 (PMC4391849; doi:10.1371/journal.pone.0123172)
Supplement: S2 Table — (DOCX) [file pone.0123172.s004.docx]

|  | MON 1 | CHA  2 | GEN  3 | LJC  4 | IC  5 | SAG  6 | RHL  7 | RPS  8 | RLA  9 | REG  10 | RBO  11 | OUE  12 | KAM  13 | FOU  14 | LOU  15 |
| --- | --- | --- | --- | --- | --- | --- | --- | --- | --- | --- | --- | --- | --- | --- | --- |
| MON 1 | 0 |  |  |  |  |  |  |  |  |  |  |  |  |  |  |
| CHA 2 | ***0,380*** | 0 |  |  |  |  |  |  |  |  |  |  |  |  |  |
| GEN 3 | ***0,235*** | 0,038 | 0 |  |  |  |  |  |  |  |  |  |  |  |  |
| LJC  4 | ***0,374*** | ***0,155*** | ***0,160*** | 0 |  |  |  |  |  |  |  |  |  |  |  |
| IC  5 | ***0,278*** | ***0,296*** | ***0,212*** | ***0,197*** | 0 |  |  |  |  |  |  |  |  |  |  |
| SAG  6 | ***0,251*** | ***0,301*** | ***0,236*** | ***0,202*** | ***0,127*** | 0 |  |  |  |  |  |  |  |  |  |
| RHL  7 | ***0,288*** | ***0,381*** | ***0,256*** | ***0,258*** | ***0,097*** | ***0,096*** | 0 |  |  |  |  |  |  |  |  |
| RPS  8 | ***0,346*** | ***0,431*** | ***0,333*** | ***0,309*** | ***0,170*** | ***0,187*** | 0,050 | 0 |  |  |  |  |  |  |  |
| RLA  9 | ***0,362*** | ***0,412*** | ***0,315*** | ***0,237*** | ***0,122*** | ***0,172*** | ***0,063*** | ***0,090*** | 0 |  |  |  |  |  |  |
| REG  10 | ***0,321*** | ***0,311*** | ***0,212*** | ***0,180*** | ***0,098*** | ***0,151*** | ***0,072*** | ***0,082*** | 0,040 | 0 |  |  |  |  |  |
| RBO  11 | ***0,341*** | ***0,411*** | ***0,205*** | ***0,229*** | ***0,123*** | ***0,261*** | ***0,148*** | ***0,121*** | 0,045 | 0,009 | 0 |  |  |  |  |
| OUE  12 | ***0,377*** | ***0,319*** | ***0,222*** | ***0,181*** | ***0,099*** | ***0,196*** | ***0,084*** | ***0,061*** | 0,039 | 0,020 | 0,011 | 0 |  |  |  |
| KAM  13 | ***0,367*** | ***0,411*** | ***0,282*** | ***0,227*** | ***0,208*** | ***0,212*** | ***0,169*** | ***0,117*** | ***0,069*** | 0,015 | 0,051 | 0,004 | 0 |  |  |
| FOU  14 | ***0,362*** | ***0,371*** | ***0,257*** | ***0,220*** | ***0,137*** | ***0,228*** | ***0,124*** | ***0,132*** | ***0,063*** | 0,038 | 0,051 | 0,020 | 0,029 | 0 |  |
| LOU  15 | ***0,370*** | ***0,428*** | ***0,307*** | ***0,278*** | ***0,149*** | ***0,211*** | ***0,127*** | ***0,080*** | 0,047 | 0,018 | 0,015 | 0,019 | 0,048 | 0,044 | 0 |
| BON  16 | ***0,355*** | ***0,425*** | ***0,291*** | ***0,260*** | ***0,197*** | ***0,252*** | ***0,173*** | ***0,184*** | ***0,100*** | ***0,084*** | 0,072 | ***0,120*** | ***0,135*** | ***0,086*** | ***0,109*** |

**S2-Table. Supplementary information.**

**F_ST_ pairwise comparisons.**

Table S2. F_ST_ pairwise comparisons based on AFLP analyses. Significant differences between sites following False Discovery Rate of 5% indicated in bold italics. Numerical IDs and sampling sites are identified in Table 2.
